# Surface TRAIL decoy receptor-4 expression is correlated with TRAIL resistance in MCF7 breast cancer cells

**DOI:** 10.1186/1471-2407-5-54

**Published:** 2005-05-25

**Authors:** Ahter D Sanlioglu, Ercument Dirice, Cigdem Aydin, Nuray Erin, Sadi Koksoy, Salih Sanlioglu

**Affiliations:** 1 The Human Gene Therapy Unit, Akdeniz University, Faculty of Medicine, Antalya, Turkey; 2 Department of Medical Biology and Genetics, Akdeniz University, Faculty of Medicine, Antalya, Turkey

## Abstract

**Background:**

Tumor Necrosis Factor (TNF)-Related Apoptosis-Inducing Ligand (TRAIL) selectively induces apoptosis in cancer cells but not in normal cells. Despite this promising feature, TRAIL resistance observed in cancer cells seriously challenged the use of TRAIL as a death ligand in gene therapy. The current dispute concerns whether or not TRAIL receptor expression pattern is the primary determinant of TRAIL sensitivity in cancer cells. This study investigates TRAIL receptor expression pattern and its connection to TRAIL resistance in breast cancer cells. In addition, a DcR2 siRNA approach and a complementary gene therapy modality involving IKK inhibition (AdIKKβKA) were also tested to verify if these approaches could sensitize MCF7 breast cancer cells to adenovirus delivery of TRAIL (Ad5hTRAIL).

**Methods:**

TRAIL sensitivity assays were conducted using Molecular Probe's Live/Dead Cellular Viability/Cytotoxicity Kit following the infection of breast cancer cells with Ad5hTRAIL. The molecular mechanism of TRAIL induced cell death under the setting of IKK inhibition was revealed by Annexin V binding. Novel quantitative Real Time RT-PCR and flow cytometry analysis were performed to disclose TRAIL receptor composition in breast cancer cells.

**Results:**

MCF7 but not MDA-MB-231 breast cancer cells displayed strong resistance to adenovirus delivery of TRAIL. Only the combinatorial use of Ad5hTRAIL and AdIKKβKA infection sensitized MCF7 breast cancer cells to TRAIL induced cell death. Moreover, novel quantitative Real Time RT-PCR assays suggested that while the level of TRAIL Decoy Receptor-4 (TRAIL-R4) expression was the highest in MCF7 cells, it was the lowest TRAIL receptor expressed in MDA-MB-231 cells. In addition, conventional flow cytometry analysis demonstrated that TRAIL resistant MCF7 cells exhibited substantial levels of TRAIL-R4 expression but not TRAIL decoy receptor-3 (TRAIL-R3) on surface. On the contrary, TRAIL sensitive MDA-MB-231 cells displayed very low levels of surface TRAIL-R4 expression. Furthermore, a DcR2 siRNA approach lowered TRAIL-R4 expression on surface and this sensitized MCF7 cells to TRAIL.

**Conclusion:**

The expression of TRAIL-R4 decoy receptor appeared to be well correlated with TRAIL resistance encountered in breast cancer cells. Both adenovirus mediated IKKβKA expression and a DcR2 siRNA approach sensitized MCF7 breast cancer cells to TRAIL.

## Background

Cancer still appears to be a challenging disease to treat. According to most recent estimates, more than 10 million new cancer cases were reported in the year 2000 killing around 6 million people [1]. In addition, 10 % of all cancers appear to be the breast cancer. Being the most frequently diagnosed cancer type in women, the breast cancer claims about 370,000 deaths each year around the world [2]. Surgery, radiotherapy and chemotherapy are among the most widely used treatment methods for patients with breast cancer [3-5]. Still, these conventional treatment modalities did not improve the survival rate of patients with locally advanced or metastatic breast cancer. With standard therapy, locally advanced breast cancer has a five year survival rate of 55 % and a ten year survival rate of 35 % [6]. There is a 40 % recurrence rate after ten years following the diagnosis and removal of primary tumor in patients with breast cancer [7]. For all these reasons, novel treatment methods are needed for the treatment of patients with breast cancer.

Induction of programmed cell death known as apoptosis [8], appears to be a viable alternative to currently employed treatment modalities in the fight against cancer [9]. In order for chemotherapy and radiotherapy treatment options to work as anticancer agents; tumor suppressor gene, p53, is required [10]. Unfortunately, p53 mutations are acquired during the progression of cancer in more than half of the human tumors [11,12]. Therefore, the resistance to both chemotherapy and radiotherapy is almost unavoidable in tumors lacking p53 [13]. On the other hand, death ligands are capable of inducing apoptosis independently of p53 status of cells [14]. Because of this reason, death ligands are currently considered as anticancer agents [15]. Among the death ligands tested, Tumor Necrosis Factor (TNF) [16-18] and FasL [19] effectively induced apoptosis in cancer cells. However, due to their systemic toxicity, the application of these agents in cancer gene therapy is very limited. The discovery of a novel death ligand, TRAIL [20,21], changed this view, since unlike other members of the TNF family, TRAIL selectively killed cancer cells without causing any harm to normal cells [22]. Thus, treating tumor cells with TRAIL ligand appeared as an invaluable way of inducing apoptosis specifically in tumor cells, as normal cells are protected against the death-inducing effects of TRAIL [23,24]. However, the mechanism of TRAIL resistance in normal cells is not understood [25] and significant proportions of cancer cells [26] including those of breast [27,28] appeared to be TRAIL resistant. Consequently, TRAIL resistance constitutes a barrier if one wishes to use TRAIL as a death ligand in any breast cancer gene therapy approach.

Resistance to TRAIL-induced apoptosis in normal cells was initially considered to be caused by the presence of decoy receptors (TRAIL-R3 and TRAIL-R4), which compete with death receptors (TRAIL-R1 and TRAIL-R2) for binding to TRAIL [29,30]. So far, no correlation between TRAIL sensitivity and the expression pattern of TRAIL receptors has been demonstrated in cancer cells yet [31]. The presence of intracellular apoptosis inhibitory substances (bcl-xL, c-FLIP, cIAP etc.) was also blamed to be responsible for TRAIL resistance [31-33]. Intriguingly, the engagement of both TRAIL death receptors and TRAIL-R4 decoy receptor also activated NF-kB pathway [24,34,35]. Because NF-kB activation is known to hamper the apoptotic pathways in cells by up-regulating the expression of various apoptosis inhibitory molecules such as cFLIP, bcl-xL, c-IAP and the decoy receptor TRAIL-R3 [34,36,37], high levels of NF-kB activation might be a strong factor responsible for blocking apoptotic processes in order to establish TRAIL resistance. For this reason, we analyzed both the TRAIL induced as well as endogenous NF-kB activities using Luciferase reporter gene assays in MCF7 breast cancer cells. Because TRAIL-R1, TRAIL-R2 and TRAIL-R4 induced NF-kB activation has been shown to be primarily mediated by TRAF2-NIK-IkappaB kinase alpha/beta signaling cascade [35], MCF7 breast cancer cells were coinfected with adenovirus vectors encoding a dominant negative mutant to IKKβ(AdIKKβKA) [38] and hTRAIL (Ad5hTRAIL) in order to test if TRAIL resistance in breast cancer cells is eliminated through the inhibition of IKK, a leading modulator of NF-kB. The molecular mechanism of TRAIL resistance in breast cancer cells (MCF7 and MDA-MB-231) was studied by novel Real Time RT-PCR assays and conventional flow cytometry in order to verify if there is any relationship between TRAIL resistance and the expression pattern of TRAIL receptors. Lastly, a DcR2 siRNA approach was utilized to knock down the expression of relevant TRAIL decoy receptor in order to reveal its connection to TRAIL resistance.

## Methods

### Recombinant adenovirus vector production

Amplification of the vectors Ad5hTRAIL [39], AdIKKβKA [17], AdEGFP [18], AdCMVLacZ [40] and AdNFkBLuc [38] was performed as previously described [41]. Amplified vectors were stored at -80°C in 10 mM Tris with 20 % glycerol. AdIKKβKA expresses a dominant negative mutant of IKKβ, which interacts with other IKK subunits to form inactive IKK complexes. The particle titers of adenoviral stocks were in the range of 10^13 ^DNA particles/ml, whereas the typical particle/plaque forming unit ratio was equal to 50.

### Infection of breast cancer cells with first generation recombinant adenovirus vectors

Breast cancer cell lines were cultured in RPMI 1640 medium supplemented with 10 % FBS, 2.2 g/l sodium bicarbonate, 1 mM L-glutamine, and 1 % penicillin-streptomycin mixture, at 37°C in a humidified 5 % CO_2 _atmosphere. Experimental steps of transduction of breast cancer cells with adenoviral vectors can be summarized as follows: Breast cancer cells were infected with an increasing multiplicity of infection (MOI) of AdEGFP (vector expressing enhanced green fluorescent protein (EGFP) reporter gene) vector at 37°C in RPMI 1640 without FBS. Two hours following infection, equal volume of RPMI 1640 supplemented with 20 % FBS was added to increase the serum concentration in the media to 10 %. 48 hours after the infection, the level of transduction was detected by examining of the percentage of GFP (+) cells under a fluorescent microscopy and subsequently by flow cytometry. Propidium iodide exclusion technique was used to determine the cell viability. Overexpression of hTRAIL was provided by Ad5hTRAIL infection. Cells were coinfected with adenovirus vectors encoding IKKβ dominant negative mutant (AdIKKβKA) and Ad5hTRAIL in order to block IKK activity thereby NF-kB activation. NF-kB promoter based Luciferase assay system was utilized to conduct NF-kB transcription activation assays using AdNFkBLuc construct. AdCMVLacZ vector was used as a control.

### NF-kB directed transcription activation assays

AdNFkBLuc construct was utilized in order to determine the NF-kB activation status of MCF7 cells. AdNFkBLuc vector [38] possesses four tandem copies of the NF-kB consensus sequence fused to a TATA-like promoter from the herpes simplex virus-thymidine kinase gene driving the expression of a Luciferase reporter. Transcriptional induction mediated by NF-kB in the presence or absence of TRAIL was measured according to the manufacturer's protocol using the Luciferase assay system with Reporter Lysis Buffer (Promega, Inc.). All measurements of Luciferase activity expressed as relative light units were normalized against the protein concentration.

### Cell viability assays

Discrimination of live cells from dead cells was performed using Live/Dead Cellular Viability/Cytotoxicity Kit from Molecular Probes (Eugene, OR). This assay is based on the use of Calsein AM and Ethidium homodimer-1 (EthD-1). Calsein AM is a fluorogenic substrate for intracellular calsein esterase. It is modified to a green fluorescent compound (calsein) by active esterase in live cells with intact membranes, thus serves as a marker for viable cells. Unharmed cell membranes do not allow EthD-1, a red fluorescent nucleic acid stain, to enter inside the cell. However, cells with damaged membrane uptake the dye and stain positive.

### Apoptosis detection by Annexin V binding

Annexin V conjugated to fluorochromes such as FITC has successfully been used as probes to detect cells undergoing apoptosis. Annexin V binding assays were carried out according to manufacturer's instructions (Alexis Biochemicals). For this purpose, a FITC conjugated mouse monoclonal antibody to human Annexin V (ALX-804-100F-T100) was employed to detect apoptotic cells via flow cytometry.

### The detection of TRAIL receptor expression profile by flow cytometry

Anti-TRAIL receptor flow cytometry set (Cat. ALX-850-273-KI01) was used to detect TRAIL receptor protein expression on cell surface. This kit contains 100μgs of MAb to TRAIL-R1 (clone HS101, Cat. 804-297A), -R2 (clone HS201, Cat.804-298A), -R3 (clone HS301, Cat. 804-344A) and -R4 (clone HS402, Cat. 804-299A). Primary antibodies were used at 5 μg/ml concentration. Biotinylated goat anti-mouse IgG1 (Cat. ALX-211-202) was used as a secondary antibody followed by streptavidin-PE (Cat. ANC-253-050) prior to flow cytometry. Flow analysis was performed according to manufacturer's protocols using BD FACSCALIBUR at the Akdeniz University Hospitals. Purified mouse IgG1 (MOPC 31C, Cat. ANC-278-010) served as an isotype control.

### Quantitative Real Time RT-PCR assay for human TRAIL receptors

TRIzol reagent (Life Technologies, Gaithersburg, MD) was used to extract total RNA from breast cancer cells, according to the instructions from the manufacturer. Reverse transcription of 2 μg of total RNA was performed using TaqMan Reverse Transcription Reagents (Applied Biosystems Cat. N8080234). Despite the fact that the sequences for TRAIL-R1 and TRAIL-R2 primers and probes were recently described by our group [42], we had to design new probe sets for the decoy receptors. Following is the sequence information for TRAIL decoy receptor sets: **TRAILR3-5' **CCC-TAA-AGT-TCG-TCG-TCG-TCA-T, **TRAILR3-3' **GGG-CAG-TGG-TGG-CAG-AGT-A, **TRAILR3 Probe: 5' **6FAM-TCGCGGTCCTGCTGCCAGTCCTAGC-TAMRA 3'; **TRAILR4-5' **ACA-GAG-GCG-CAG-CCT-CAA, **TRAILR4-3' **ACG-GGT-TAC-AGG-CTC-CAG-TAT-ATT, **TRAILR4 Probe: 5' **6FAM-AGGAGGAGTGTCCAGCAGGATCTCATAGATC-TAMRA 3'. rRNA was amplified as an internal control in the same reaction. Both the rRNA primers and probes were obtained from PE Applied Biosystems (Cat. 4308329). ΔΔCt method was used as described by Applied Biosystems to calculate the relative quantities of TRAIL receptors. The TaqMan PCR reaction was performed as described by the manufacturer (Applied Biosystems Cat. N8080228).

### A DcR2 siRNA approach targeting TRAIL-R4 expression

Posttranscriptional silencing of gene expression became a very useful approach within the last couple of years in research. DcR2 siRNA experiments were performed using DcR2 siRNA (sc-35185), siRNA transfection medium (sc-36868) and siRNA transfection reagent (sc-29528) in MCF7 breast cancer cells as described by the manufacturer (Santa Cruz Biotechnology). Flow cytometry analysis was performed to assess any changes in TRAIL-R4 gene expression. MCF7 cells were infected with Ad5hTRAIL or AdCMVLacZ vectors at increasing doses 35 hours following the transfection. Molecular Probe's Live/Dead Cellular Viability/Cytotoxicity Kit was used to assess the amount of live cells 48 hours following the infection.

## Results

### MCF7 breast carcinoma cells were efficiently transduced with recombinant adenoviruses

**Figure 1:**
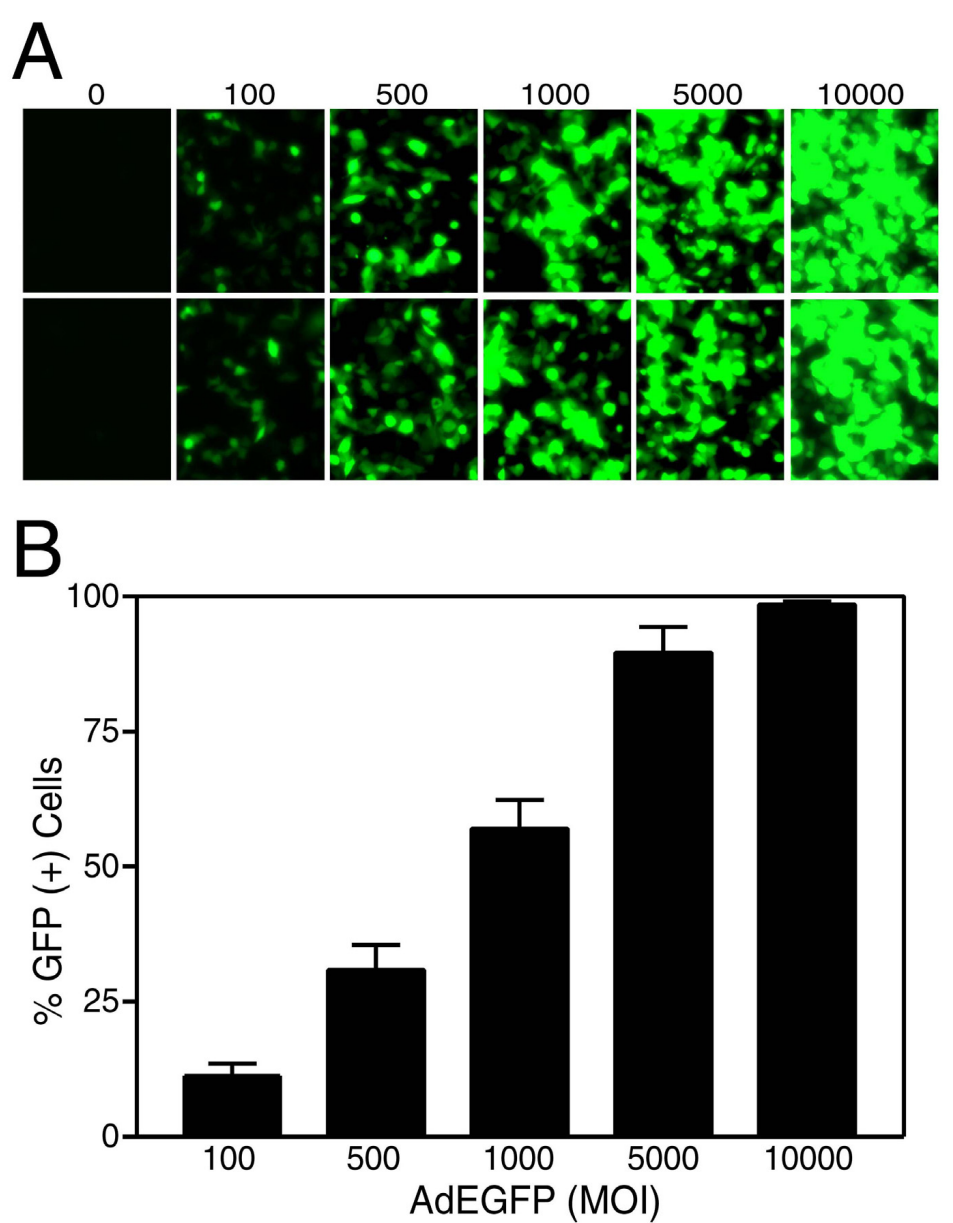
First generation adenoviral vectors efficiently transduced MCF7 breast cancer cells. MCF7 cells were infected with increasing MOIs of AdEGFP for 48 hours prior to analysis. The number of EGFP expressing cells was detected under fluorescent microscopy (Panel A), and analyzed by flow cytometry (Panel B). Numbers represent viral doses applied in MOI values as DNA particles/cell.

In order to find out the efficacy of transduction of breast cancer cells by first generation adenoviral vectors, MCF7 cells were infected with increasing Multiplicity of Infection (MOI) of adenovirus encoding Enhanced Green Fluorescent Protein (AdEGFP). The transduction profiles were followed under fluorescent microscopy and the results were quantitatively analyzed by flow cytometry 48 hours following the infection (Figure 1). While an MOI of 5000 DNA particles/cell was sufficient to transduce more than 90 % of the cells, nearly 100 % of the cells were transduced with AdEGFP at an MOI of 10,000 DNA particles/cell. These assays were also pivotal in obtaining the optimum dose of adenovirus required for efficient transduction of MCF7 breast carcinoma cell line without observing deleterious cytotoxic effects. These results demonstrated that breast cancer cells were transduced successfully with recombinant adenoviral vectors.

### MCF7 breast cancer cells displayed complete resistance to TRAIL

**Figure 2:**
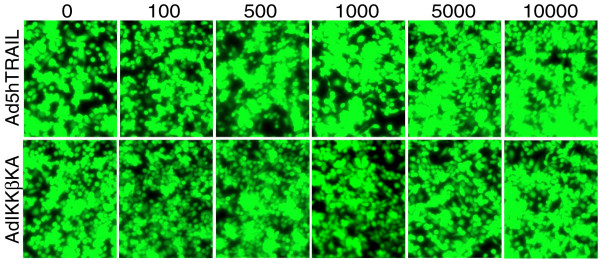
Ad5hTRAIL or AdIKKβKA infection alone did not decrease the viability of MCF7 breast cancer cells. MCF7 cells were infected with increasing MOIs of either Ad5hTRAIL or AdIKKβKA construct. Cell viability was detected using Molecular Probe's Live/Dead Cellular Viability/Cytotoxicity Kit 48 hours following the infection. Numbers represent viral doses applied, in MOI values as DNA particles/cell.

Although TRAIL appeared as a promising therapeutic ligand to treat cancer, a variety of tumor types were reported to be resistant to TRAIL-induced cell death. For this reason, we wanted to investigate if exogenous TRAIL expression delivered by adenovirus vectors would induce killing of breast cancer cells. To test this, MCF7 cells were infected with increasing titers of Ad5hTRAIL or AdCMVLacZ. Amount of viable cells were detected using Molecular Probe's Live/Dead Cellular Viability/Cytotoxicity Kit 48 hours following the infections (Figure 2). MCF7 cells displayed complete resistance to TRAIL, as no reduction in the level of viable cells was observed even at an MOI of 10,000 DNA particles/cell, at which almost all cells were infected. Thus, it was concluded that MCF7 breast cancer cells were completely resistant to adenovirus delivery of TRAIL. Similarly, AdCMVLacZ infection alone revealed no significant degree of cell death either (data not shown).

### Blocking IKK induced NF-kB activation pathway alone did not cause any reduction in the viability of MCF7 breast carcinoma cells

Because increased NF-kB activity was claimed to be responsible for the resistance to death ligand induced cytotoxicity in some tumors [36,37], we wanted to test if the inhibition of IKK activity thereby NF-kB would reduce the viability of breast cancer cells. In order to block the intracellular anti-apoptotic NF-kB pathway, MCF7 cells were infected with increasing MOIs of adenoviral vectors encoding a dominant negative mutant of IKKβ(AdIKKβKA), a key molecule involved in the activation of NF-kB. Cell viability was examined 48 hours following the infection under fluorescent microscope (Figure 2). Interestingly, AdIKKβKA vector alone proved inefficient in reducing the viability of MCF7 cells, even at an MOI of 10,000 DNA particles/cell.

### Adenovirus delivery of IKKβKA gene expression sensitized MCF7 breast cancer cells to TRAIL-induced apoptosis

**Figure 3:**
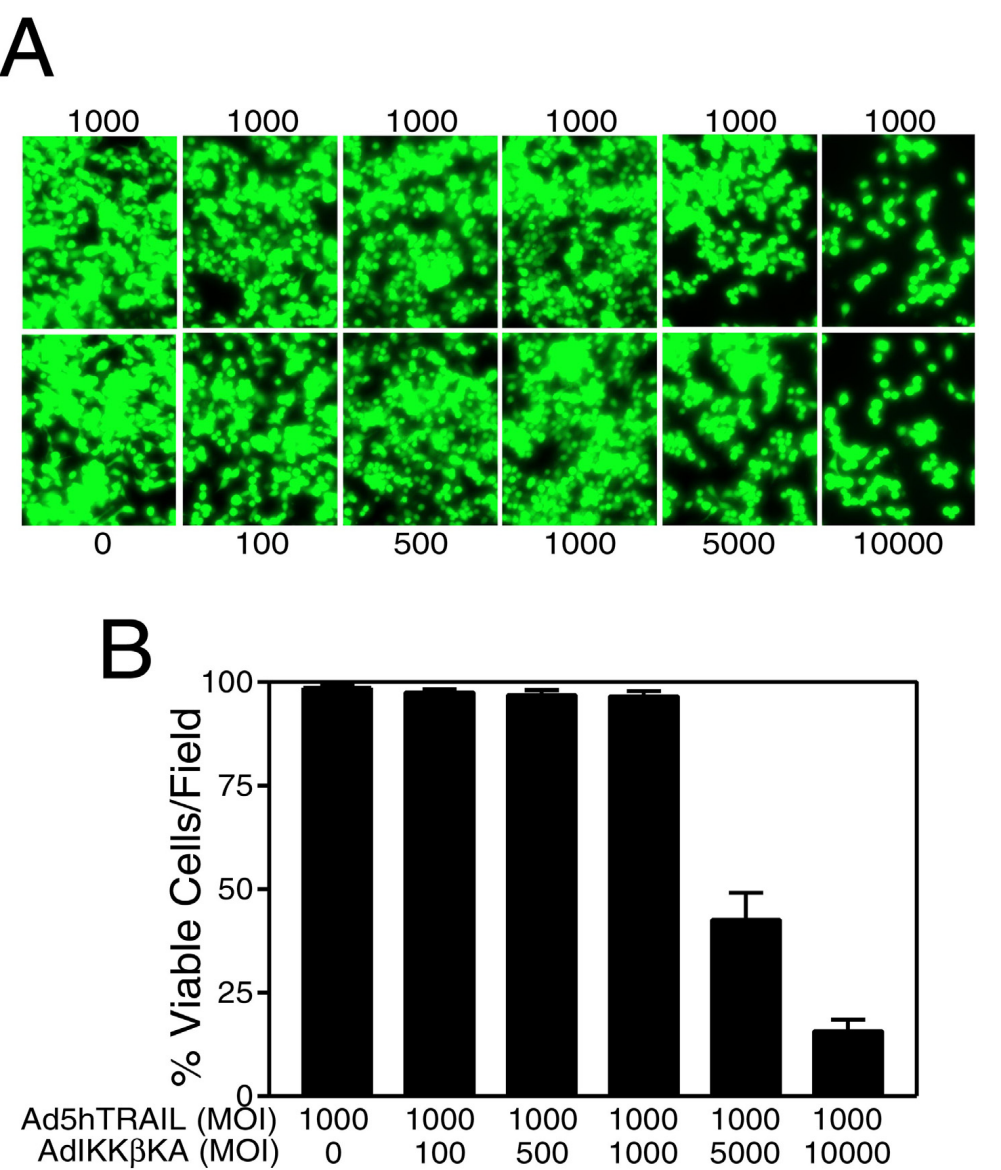
IKKβKA expression via adenoviral vectors sensitized MCF7 cells to TRAIL-mediated apoptosis. MCF7 cells were infected with increasing doses of adenoviral vectors encoding dominant negative mutant of IKKβ (as shown below each panel), while simultaneous infection with Ad5hTRAIL (as shown above each panel) was performed at a constant MOI of 1000. Cell viability was detected using Molecular Probe's Live/Dead Cellular Viability/Cytotoxicity Kit 48 hours following infection. Numbers represent viral doses applied in MOI values as DNA particles/cell. Fluorescent micrographs are provided in Panel A; Panel B depicts quantitative analysis of such infections. Values represent the mean (± SEM) of three different experiments.

**Figure 4:**
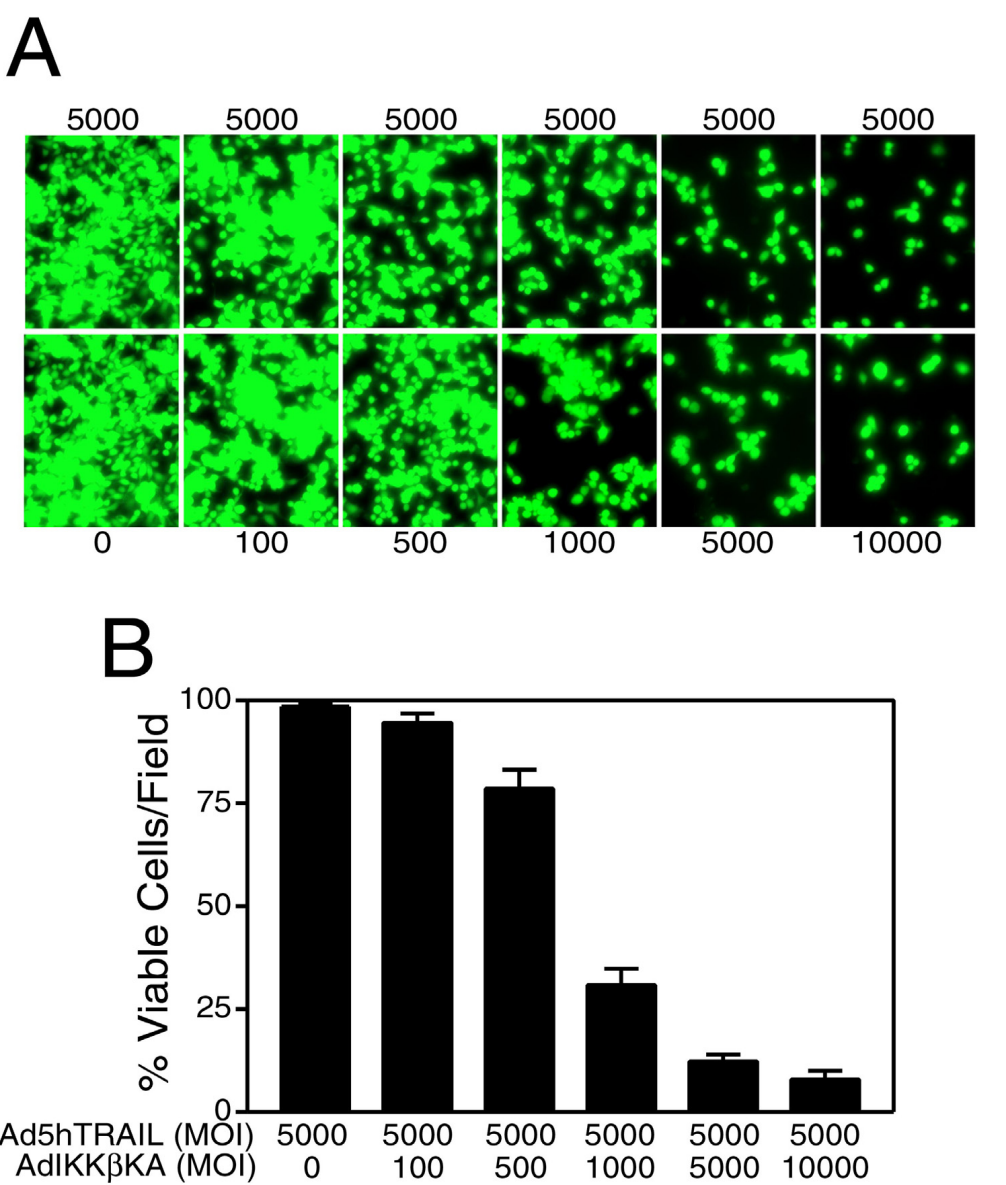
AdIKKβKA infection defeated the resistance to TRAIL-induced apoptosis in MCF7 breast cancer cells. These cells were coinfected with a constant MOI of 5000 DNA particles/cell of Ad5hTRAIL (as shown above each panel) and increasing doses of AdIKKβKA (as shown below each panel). Live/Dead Cellular Viability/Cytotoxicity Kit from Molecular Probe was used to detect TRAIL cytotoxicity 48 hours following infection. Numbers represent viral doses applied, in MOI values as DNA particles/cell. Data represent the mean of (± SEM) six independent data points (n = 6).

Adenovirus-mediated delivery of IKKβ (Ad.IKKβKA) [17,18] or IkBα (Ad.IkBαSR) [40,43] dominant negative mutants have previously been demonstrated to sensitize lung cancer cells to TNF death ligand. Because most of the breast cancer cell lines tested appeared to be TRAIL resistant [27,28], NF-kB targeting strategies involving IKK inhibition was employed to verify whether MCF7 breast carcinoma cells were sensitized to TRAIL under these circumstances. To accomplish this, MCF7 cells were coinfected with a constant MOI of Ad5hTRAIL construct and increasing doses of AdIKKβKA vector. In order to better assess the sensitization phenomenon, Ad5hTRAIL was infected at two different MOIs into MCF7 breast cancer cell lines. While a constant MOI of 1000 DNA particles/cell of Ad5hTRAIL was used in infection experiments depicted on Figure 3, infection experiments conducted at an MOI of 5000 DNA particles/cell are displayed in Figure 4. The amount of viable cells was detected 48 hours following the infections using Molecular Probe's Live/Dead Cellular Viability/Cytotoxicity Kit. Intriguingly, MCF7 cells were sensitized to TRAIL only when Ad5hTRAIL was coinfected with AdIKKβKA vector. For instance, nearly 55 % cell death was observed when cells were coinfected with 1000 MOI of Ad5hTRAIL and 5000 MOI of AdIKKβKA constructs (Figure 3). When MOI of Ad5hTRAIL was increased to 5000 as depicted on Figure 4, the death rate went up to 90 %. On the other hand, AdCMVLacZ infection instead of AdIKKβKA in breast cancer cells revealed no TRAIL sensitization (data not shown). These results suggested that IKKβKA expression via adenoviral vectors defeated TRAIL resistance observed in MCF7 breast cancer cells.

### Exogenous TRAIL overexpression elevated the basal NF-kB activity in MCF7 cells, whereas IKKβKA expression blocked both TRAIL-induced and basal NF-kB activities

**Figure 5:**
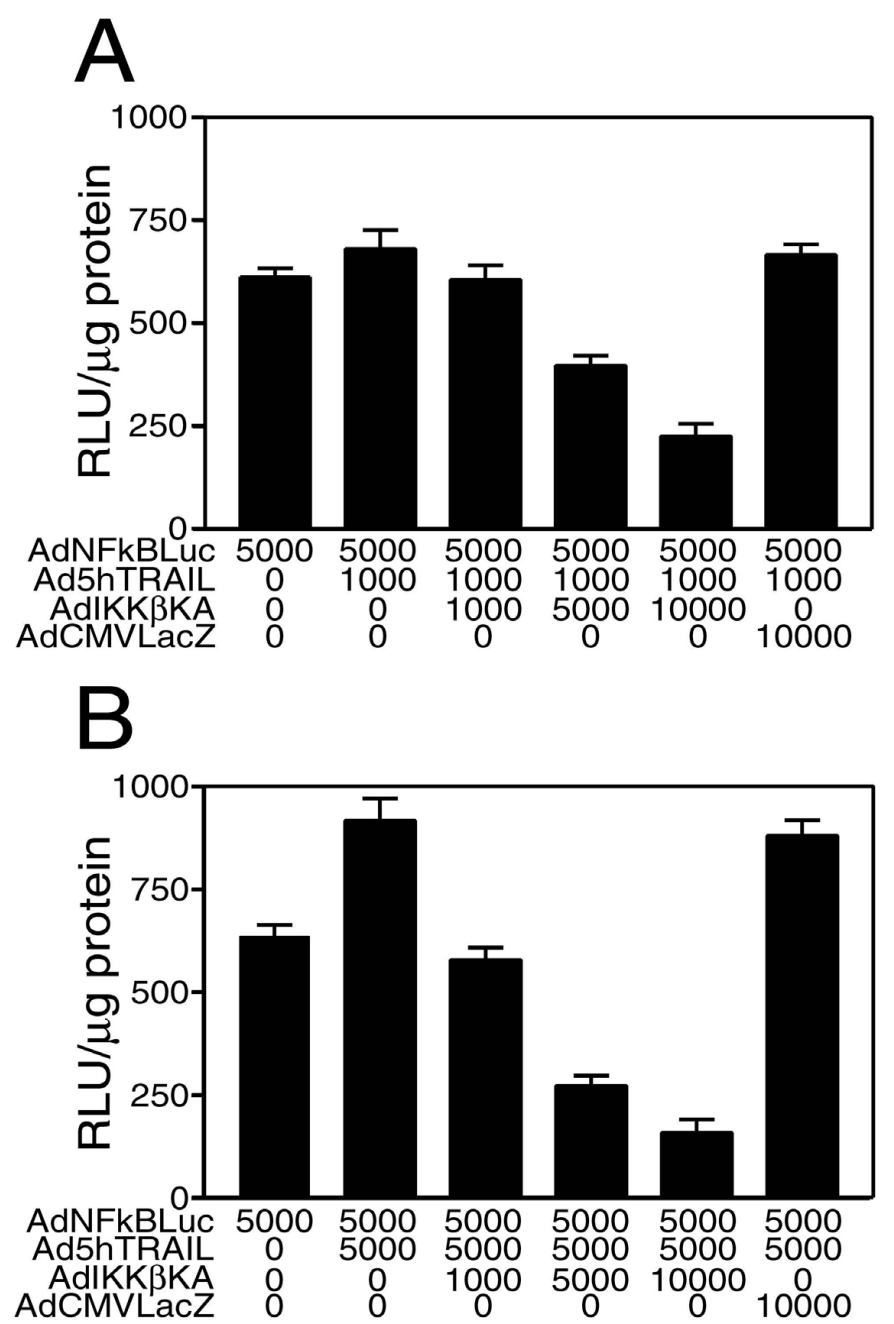
Distinctive regulation of NF-kB activation in MCF7 breast cancer cells by Ad5hTRAIL and/or AdIKKβKA infections. MCF7 cells were simultaneously infected with AdNFkBLuc, Ad5hTRAIL and/or increasing doses of AdIKKβKA construct for 24 hours. AdCMVLacZ infection was also performed as a negative control. The types of constructs used in the infection are shown on the x axis. MOI values represent DNA particles/cell. Ad5hTRAIL vector was used at two different constant MOIs (MOI of 1000 and 5000) in order to avoid cell death complicating our assay result. Luciferase activity expressed in Relative Light Units per microgram protein is shown on y axis. Values represent the mean (± SEM) of six independent data points (n = 6).

It is well known that different tumor cells display diverse levels of endogenous NF-kB activities. Furthermore, intracellular NF-kB activity in tumor cells is upregulated by both TRAIL death receptors (TRAIL-R1 and TRAIL-R2) [34,44] as well as TRAIL decoy receptor TRAIL-R4 [45] upon ligand binding. Knowing the endogenous NF-kB status of cancer cells before the therapy is obviously crucial for TRAIL mediated gene therapy targeting to induce apoptosis in cancer cells. A coinfection experiment was performed using a recombinant adenovirus vector carrying NF-kB driven Luciferase reporter gene (AdNFkBLuc) and Ad5hTRAIL vector in order to study the extent of NF-kB activation as a result of TRAIL overexpression in MCF7 breast cancer cell line. NF-kB Luciferase assays were conducted 24 hours following the infection in order to determine cell's NF-kB activation status. As seen in Figure 5, Ad5hTRAIL at an MOI of 5000 DNA particles/cell (Panel B) but not at an MOI of 1000 DNA particles/cell (Panel A) stimulated NF-kB activation. In order to determine the magnitude of NF-kB inhibition, a triple coinfection experiment involving AdNFkBLuc, Ad5hTRAIL and AdIKKβKA or AdCMVLacZ was performed. While IKKβKA overexpression in MCF7 cells gradually reduced both the TRAIL-induced and basal NF-kB activities in MCF7 cells, no such NF-kB inhibiting effect was observed in cells upon super-infection with AdCMVLacZ virus as a control (Figure 5).

### Coinfection of Ad5hTRAIL and AdIKKβKA results in apoptotic cell death in MCF7 breast cancer cells

**Figure 6:**
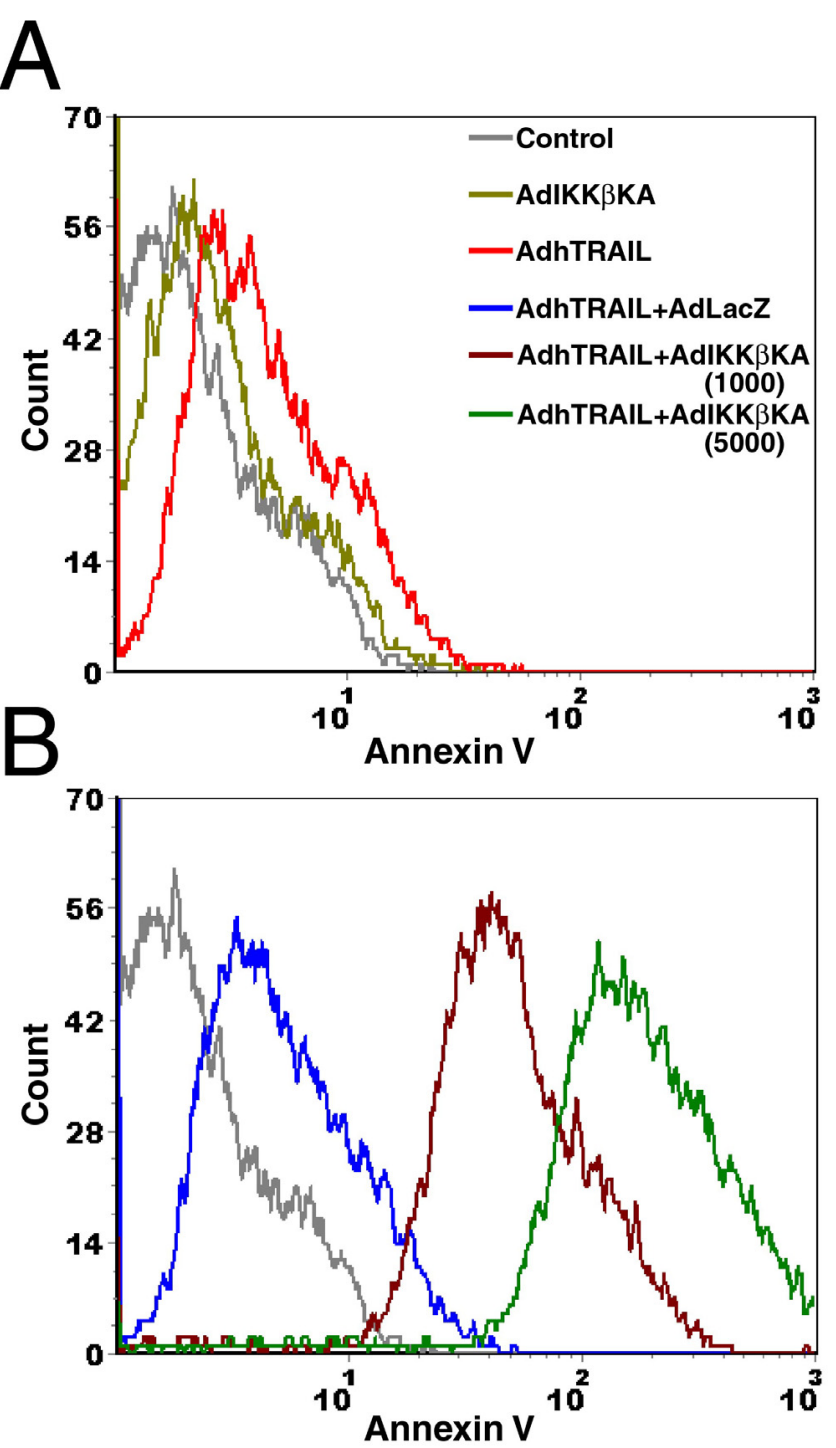
Ad5hTRAIL and AdIKKβKA coinfection induced apoptosis in MCF7 breast carcinoma cells. FITC conjugated Annexin V and Propidium Iodide (PI) staining were utilized using MCF7 cells infected with various combinations of adenovirus constructs as described in Methods prior to flow cytometry. Each histogram represents 10^4 ^gated MCF7 cells. Histograms were illustrated in two panels for clarity. Various treatment settings were provided in Panel A. MOI of 5000 DNA particles/cell was used for each viral construct unless stated otherwise in the Figure. Control line represents uninfected but FITC-Annexin V and PI stained MCF7 cells. Only one representative assay out of three independent assays was provided.

To show that apoptosis is the mechanism of cell death mediated by TRAIL overexpression under the setting of IKK inhibition in MCF7 cells, Annexin V staining was performed using flow cytometry. For this purpose, MCF7 cells were infected with Ad5hTRAIL or AdIKKβKA vectors alone or in combination. Thirty-five hours following the infection, apoptotic cell death was analyzed by Annexin-V-FITC staining. As displayed in Figure 6 Panel A, there was no substantial Annexin V binding generated by the expression of TRAIL or IKKβKA in MCF7 cells. However, considerable levels of Annexin V binding were observed in cells coinfected with Ad5hTRAIL and AdIKKβKA indicating apoptotic cell death (Figure 6, Panel B). As predicted, Ad5hTRAIL and AdCMVLacZ (negative control) coinfection did not yield any significant levels of Annexin V binding as MCF7 cells are resistant to TRAIL in the absence of IKK inhibition. These results suggested that the mechanism of cell death experienced by MCF7 cells is apoptosis following TRAIL stimulation under the setting of IKK inhibition.

### MCF7 breast cancer cell line displayed significant levels of TRAIL decoy receptor-4 expression

**Figure 7:**
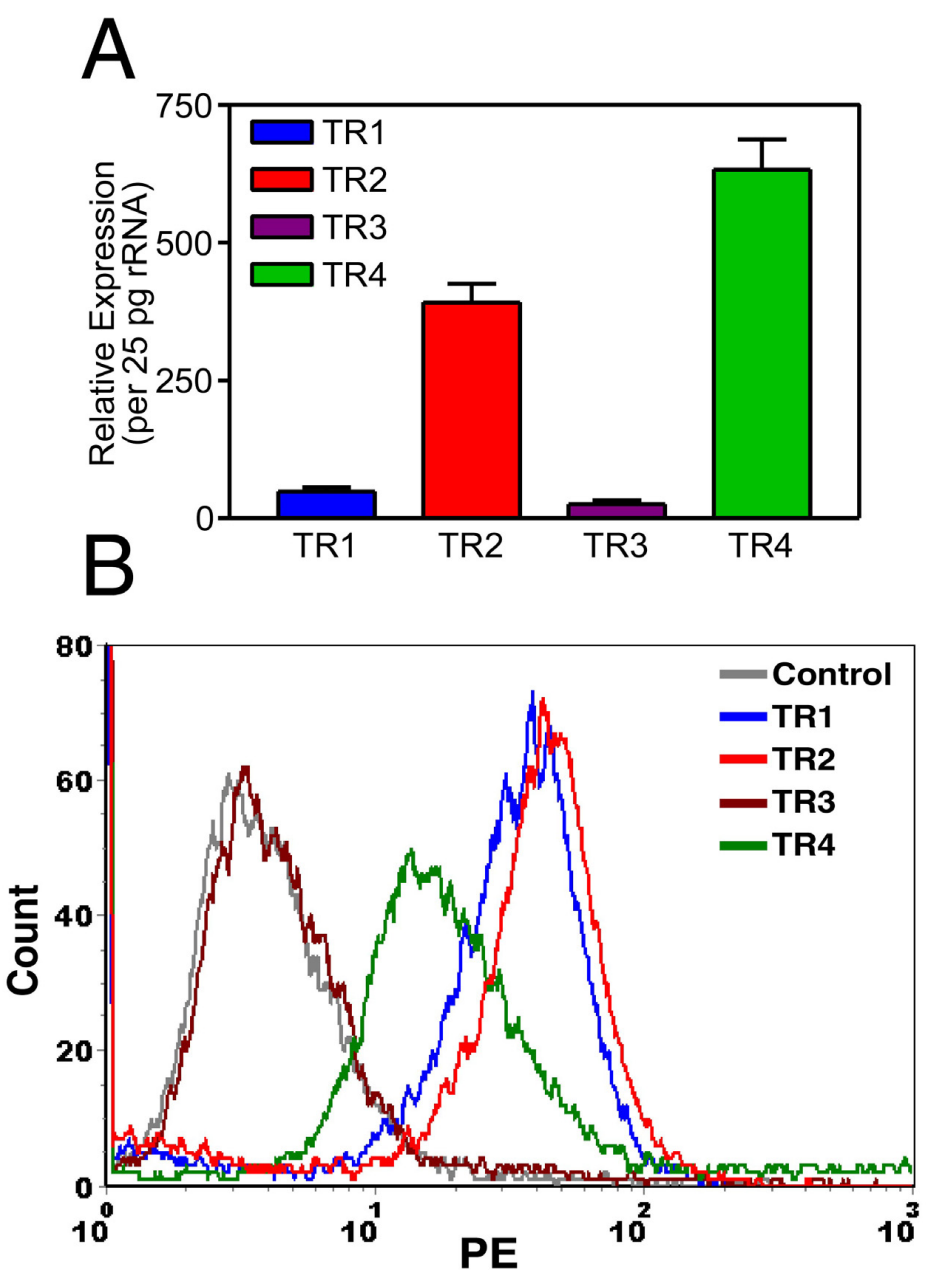
MCF7 breast carcinoma cell line displayed substantial levels of TRAIL-R4 decoy receptor expression. Quantitative Real Time RT-PCR of TRAIL receptors was performed as described in Methods (Panel A). TRAIL receptor levels per 25 pg of ribosomal cDNA are presented in the graph for clarity. Ribosomal RNA primers and probes were included in each TaqMan reaction as an internal control. Panel B depicts the surface TRAIL receptor expression pattern of MCF7 cells using flow cytometry. Experimental parameters are defined in colored lines. 10^4 ^cells were gated for each histogram. Only one representative assay for each experiment (independently repeated three times) is shown.

So far no evidence of the connection between the expression pattern of TRAIL receptors and TRAIL sensitivity was found in cancer cells [31]. Part of the reason might have been the inability to screen all TRAIL receptors at once in breast cancer cells then [28]. In order to compensate this deficiency, quantitative novel Real Time RT-PCR assays were conducted using primer-probe sets specifically designed to detect each TRAIL receptor in MCF7 breast cancer cells (Figure 7, Panel A). According to our results, while all TRAIL receptors were expressed in MCF7 cells, TRAIL-R4 expression was the highest among the four. In addition, the level of TRAIL-R2 expression was much higher than that of TRAIL-R1. Lastly, TRAIL-R3 decoy receptor expression was the lowest. These results suggested that high levels of TRAIL-R4 decoy receptor expression correlated well with TRAIL resistance. However, as the gene expression detected inside the cell may not necessarily correlate with the receptor expression on cell surface, we decided to perform flow cytometry analysis using antibodies specific to four different TRAIL receptors. As shown in Figure 7 Panel B, MCF7 cells expressed all TRAIL receptors excluding TRAIL-R3 on cell surface. While similar levels of TRAIL death receptors TRAIL-R1 and TRAIL-R2 were expressed, there were still considerable levels of TRAIL-R4 decoy receptor expression on the surface of MCF7 cells.

### TRAIL sensitive MDA-MB-231 cells displayed very low levels of TRAIL-R4 decoy receptor expression on cell surface

**Figure 8:**
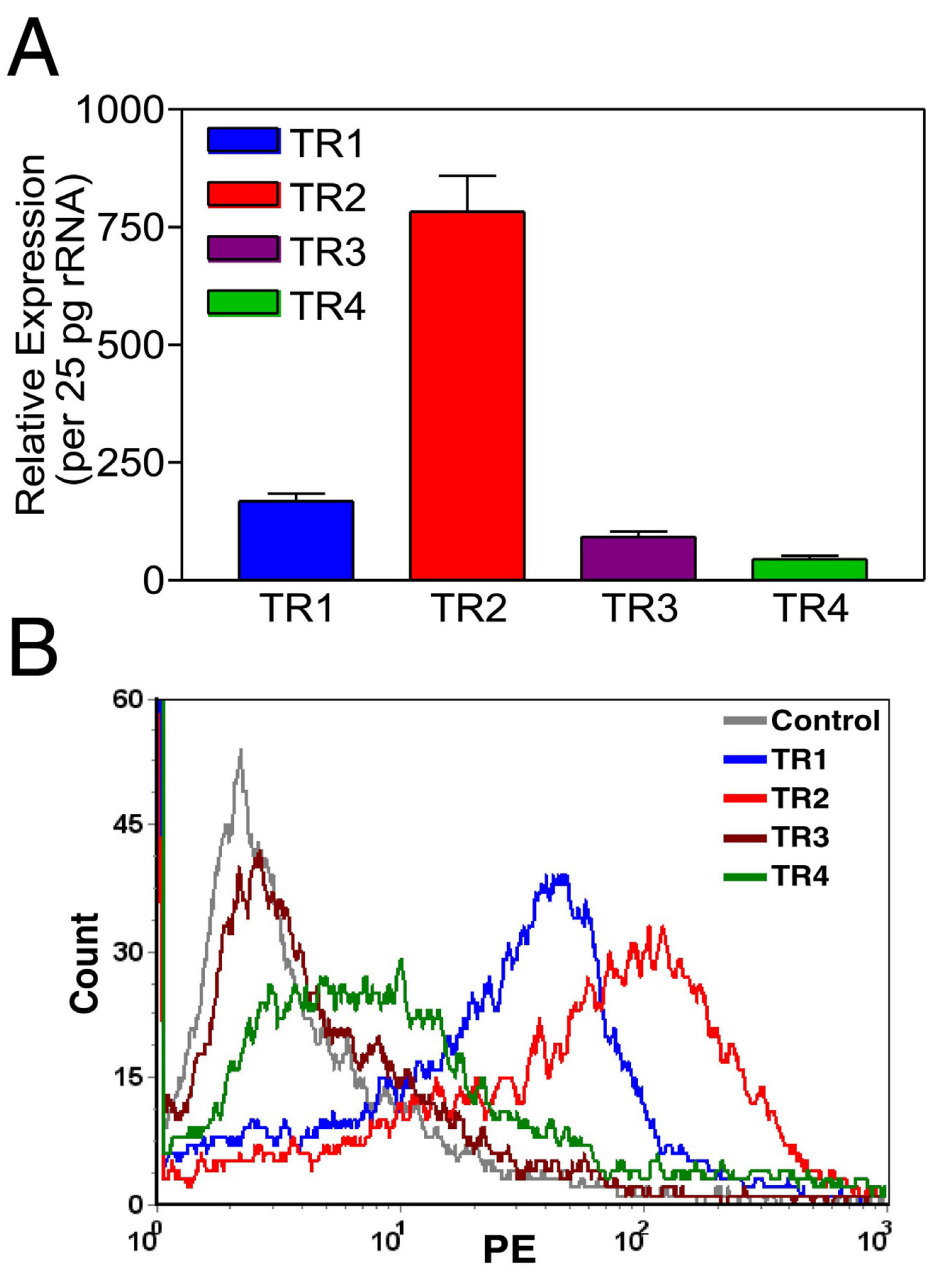
MDA-MB-231 breast cancer cells displayed trivial levels of TRAIL-R4 decoy receptor expression on surface. TRAIL receptor composition of MDA-MB-231 breast cancer cells revealed by Real Time RT-PCR assay is displayed in Panel A. Panel B illustrates flow cytometry analysis showing the surface expression pattern of TRAIL receptors. 10^4 ^cells were gated for each histogram. Only one representative assay out of three is shown.

**Figure 9:**
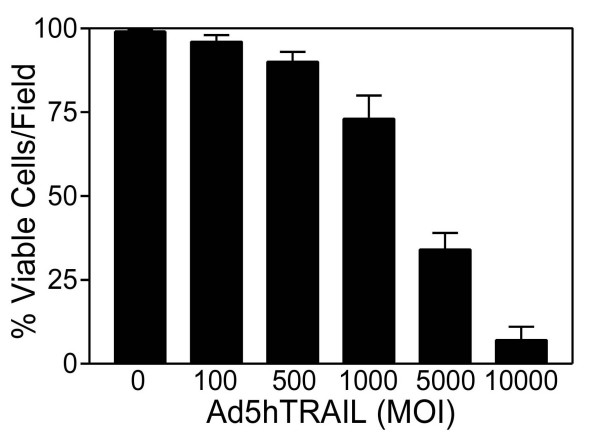
MDA-MB-231 breast cancer cell line is sensitive to Ad5hTRAIL infection. MDA-MB-231 breast cancer cells were infected with increasing MOIs of Ad5hTRAIL construct. Molecular Probe's Live/Dead Cellular Viability/Cytotoxicity Kit was used to detect % viable cells 48 hours following the infection. Numbers represent viral doses applied in MOI values as DNA particles/cell. Values represent the mean (± SEM) of six independent data points (n = 6).

In order to solidify the importance of TRAIL-R4 expression and its connection to TRAIL resistance, another breast cancer cell line, MDA-MB-231, was also analyzed in terms of TRAIL receptor expression profile. Real Time RT-PCR assays revealed that while TRAIL-R2 expression was the highest on transcript levels, TRAIL-R4 decoy receptor expression was the lowest TRAIL receptor expressed in MDA-MB-231 breast cancer cells (Figure 8, Panel A). Furthermore, flow cytometry analysis indicated that insignificant levels of TRAIL-R4 expression were detected on the surface of MDA-MB-231 breast cancer cells (Figure 8, Panel B). TRAIL-R3 decoy receptor expression, however, was not detectable using flow cytometry. Intriguingly, in contrast to what was observed with MCF7, adenovirus delivery of TRAIL alone killed significant proportions of MDA-MB-231 breast cancer cells (Figure 9).

### Lowering of TRAIL-R4 gene expression sensitized MCF7 breast cancer cells to TRAIL

**Figure 10:**
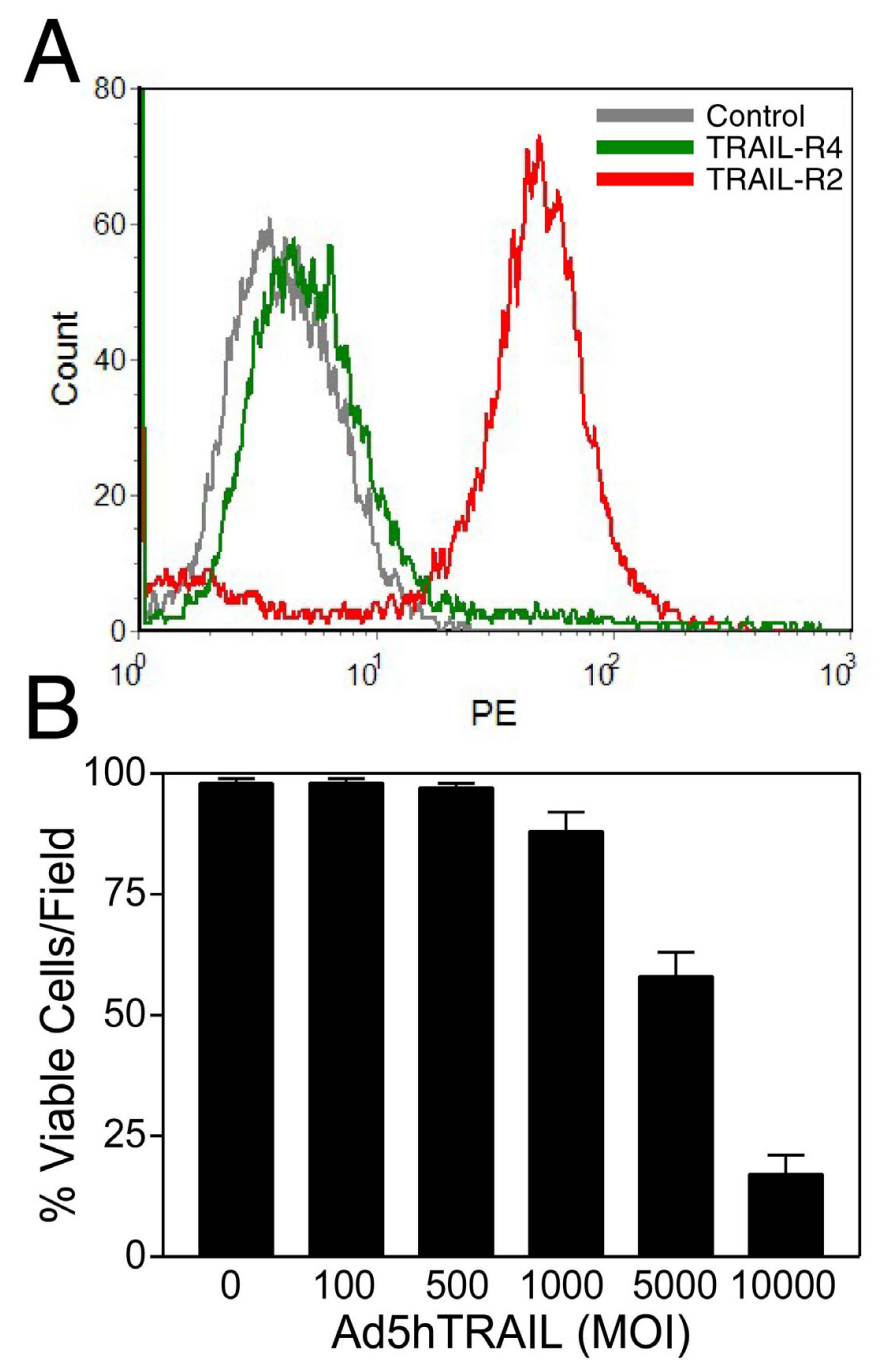
Knocking down TRAIL-R4 expression sensitized MCF7 breast cancer cells to TRAIL. A DcR2 siRNA approach was administered as described in Methods using TRAIL resistant MCF7 breast cancer cell line. Panel A depicts a flow cytometry analysis confirming strong attenuation of TRAIL-R4 expression on cell surface. TRAIL-R2 death receptor expression was also detected as a control. Sensitization of MCF7 breast cancer cells to TRAIL following a DcR2 siRNA approach is provided in Panel B. MCF7 breast cancer cells were infected with increasing doses of Ad5hTRAIL alone following a DcR2 siRNA transfection. Cell death was detected 48 hours following the infection (Panel B). Data represent the mean (± SEM) of 6 independent data points.

In order to solidify the connection between TRAIL-R4 decoy receptor gene expression and TRAIL resistance, a DcR2 siRNA approach was executed in TRAIL resistant MCF7 breast cancer cells. Flow cytometry analysis conducted 35 hours following the transfection revealed that the level of TRAIL-R4 protein expression on surface went down drastically (Figure 10, Panel A). At this stage, MCF7 cells were further infected with either Ad5hTRAIL or AdCMVLacZ vector at increasing doses. Cell viability assays were conducted 48 hours following the infection. Only Ad5hTRAIL infected cells exhibited considerable amount of cell death following transfection (Figure 10, Panel B). No such effect was observed when cells were infected with AdCMVLacZ virus (data not shown).

## Discussion

Although, conventional treatment modalities could not satisfactorily improve the survival rates of patients with locally advanced and metastatic disease, adenovirus delivery of death ligands represents a feasible choice for the treatment of patients with breast cancer. However, recent observations demonstrating that a considerable portion of human cancers including those of the breast [27,28] were TRAIL resistant undermined the potential application of TRAIL against cancer. Accordingly, the understanding of the mechanism of TRAIL resistance is the key to resolve primary obstacles in TRAIL mediated gene therapy approach. Based on recent findings from our laboratory and others, we think that NF-kB signaling is one of the most crucial pathways involved in the constitution of TRAIL resistance [26]. Despite the fact that TRAIL-R1, TRAIL-R2 and TRAIL-R4 induced NF-kB activation has been shown to be primarily mediated by TRAF2-NIK-IkappaB kinase alpha/beta signaling cascade [35], there is some doubt on whether or not NF-kB activation can block TRAIL mediated apoptosis. For example, in one particular study it was reported that NF-kB inhibition by way of IkappaBalpha mutant expression sensitized MCF7 cells to TNF but not TRAIL-induced apoptosis [35]. Considering the fact that there are different ways to activate NF-kB pathway (IkB dependent and independent ways) [46] we decided to inhibit IKK activity rather than targeting IkappaBalpha itself to look for the possibility of sensitizing MCF7 breast cancer cells to TRAIL.

First of all, in order to find out the efficacy of adenovirus transduction in breast cancer cells, MCF7 cells were infected with increasing MOIs of AdEGFP virus. The transduction profiles analyzed by flow cytometry showed that nearly 100 % of the cells were transduced with AdEGFP at an MOI of 10,000 DNA particles/cell (Figure 1). The efficacy of TRAIL in mediating apoptosis of MCF7 breast cancer cells was assessed using Ad5hTRAIL construct. Interestingly, MCF7 cells displayed complete resistance to TRAIL as no reduction in the level of viable cells was observed even at an MOI of 10,000 DNA particles/cell (Figure 2). IKK inhibiting strategy alone proved inefficient in reducing the viability of MCF7 cells suggesting that an apoptotic stimulus was required in order to induce cell killing (Figure 2). Interestingly, in order to break down TRAIL resistance and to induce cell death, a coinfection of MCF7 cells with Ad5hTRAIL and AdIKKβKA was required (Figures 3 and 4). Luciferase assays confirmed that both the TRAIL induced and endogenous NF-kB activities were drastically reduced by the infection of MCF7 cells with AdIKKβKA virus (Figure 5). Moreover, IKKβKA sensitization of MCF7 breast carcinoma cells resulted in TRAIL induced apoptosis as revealed by Annexin V binding assays (Figure 6). These results suggested that NF-kB activation pathway has a hampering effect on TRAIL-induced cell death in MCF7 cells, and blocking this pathway is essential to sensitize breast cancer cells to TRAIL mediated apoptosis.

So far, no correlation between TRAIL resistance and TRAIL decoy receptor gene expression has been reported. For example, analysis of breast cancer cell lines by just examining the expression levels of TRAIL death receptors (TRAIL-R1 and TRAIL-R2) and TRAIL-R3 decoy receptor using RNase protection assay did not reveal any connection between the expression pattern of TRAIL receptors and TRAIL resistance [28]. But whether or not TRAIL-R4 decoy receptor gene expression in any way contributes to TRAIL resistance in breast cancer cells remains to be tested yet. Quantitative Real Time RT-PCR assays were developed in order to assess the level of TRAIL receptor gene expression in breast carcinoma cells. While all TRAIL receptors were detectable in MCF7 breast carcinoma cell line, the level of TRAIL-R4 decoy receptor gene expression was the highest among the four (Figure 7, Panel A). This intriguing observation is consistent with a previous report suggesting that transient TRAIL-R4 overexpression protected target cells from TRAIL induced cytotoxicity [45]. TRAIL R4 is known to protect cells from apoptosis by acting both as a decoy receptor and an antiapoptotic signal provider. While Real Time PCR assay is useful in assessing the level of gene expression on mRNA levels, obviously this assay does not necessarily reflect TRAIL receptor composition on cell surface. For this reason, conventional flow cytometry analysis was carried out in order to determine the level of TRAIL receptor protein expression on cell surface. Despite the presence of TRAIL death receptors, substantial levels of TRAIL-R4 decoy receptor expression were detectable on the surface of MCF7 breast carcinoma cells (Figure 7, Panel B). On top of that, TRAIL sensitive MDA-MB-231 cell line (Figure 9) displayed very low levels of TRAIL-R4 decoy receptor expression on cell surface (Figure 8, Panel B). Neither of the cell lines expressed detectable levels of TRAIL-R3 decoy receptor on surface. Intriguingly, administration of a DcR2 siRNA approach lowered surface TRAIL-R4 expression and sensitized MCF7 breast cancer cells to TRAIL (Figure 10).

## Conclusion

Our results demonstrated that the expression of TRAIL-R4 decoy receptor but not TRAIL-R3 appeared to correlate well with TRAIL resistance phenotype observed in MCF7 breast cancer cells. Further screening of another breast cancer cell line, MDA-MB-231, revealed that low levels of TRAIL-R4 expression on surface were correlated with TRAIL sensitivity. These results strengthen our argument that TRAIL-R4 but not TRAIL-R3 is the decoy receptor which appeared to influence TRAIL sensitivity in breast cancer cells. This is further confirmed by a DcR2 siRNA assay which suggested that down regulation of TRAIL-R4 expression sensitized MCF7 breast cancer cells to TRAIL. In addition, the inhibition of IKK pathway thereby NF-kB sensitized MCF7 cells to TRAIL induced apoptosis despite the expression of TRAIL-R4 decoy receptor on cell surface. Consequently, this complementary gene therapy approach involving IKK inhibition might be necessary to breakdown TRAIL resistance encountered in patients with breast cancer.

## Abbreviations

TRAIL= Tumor Necrosis Factor (TNF)-Related Apoptosis-Inducing Ligand, EGFP= Enhanced Green Fluorescent Protein, MOI= Multiplicity of Infection, DcR2= Decoy receptor 2.

## Competing interests

The author(s) declare that they have no competing interests.

## Authors' contributions

ADS performed cell viability, Luciferase, Flow Cytometry, Real Time RT-PCR and siRNA assays, ED assisted ADS with adenovirus preparation, CA performed AdEGFP transduction assays, NE cultured breast cancer cells, SK optimized flow cytometry assays, SS participated in the coordination and execution of the study. All authors read and approved the final manuscript.

## Pre-publication history

The pre-publication history for this paper can be accessed here:


